# Enhancing Wearable Fall Detection System via Synthetic Data

**DOI:** 10.3390/s25154639

**Published:** 2025-07-26

**Authors:** Minakshi Debnath, Sana Alamgeer, Md Shahriar Kabir, Anne H. Ngu

**Affiliations:** Department of Computer Science, Texas State University, San Marcos, TX 78666-4684, USA; stg60@txstate.edu (M.D.); sana.alamgeer@txstate.edu (S.A.); cpi12@txstate.edu (M.S.K.)

**Keywords:** fall detection, time-series data, synthetic data generation, Diffusion, video extraction

## Abstract

Deep learning models rely heavily on extensive training data, but obtaining sufficient real-world data remains a major challenge in clinical fields. To address this, we explore methods for generating realistic synthetic multivariate fall data to supplement limited real-world samples collected from three fall-related datasets: SmartFallMM, UniMib, and K-Fall. We apply three conventional time-series augmentation techniques, a Diffusion-based generative AI method, and a novel approach that extracts fall segments from public video footage of older adults. A key innovation of our work is the exploration of two distinct approaches: video-based pose estimation to extract fall segments from public footage, and Diffusion models to generate synthetic fall signals. Both methods independently enable the creation of highly realistic and diverse synthetic data tailored to specific sensor placements. To our knowledge, these approaches and especially their application in fall detection represent rarely explored directions in this research area. To assess the quality of the synthetic data, we use quantitative metrics, including the Fréchet Inception Distance (FID), Discriminative Score, Predictive Score, Jensen–Shannon Divergence (JSD), and Kolmogorov–Smirnov (KS) test, and visually inspect temporal patterns for structural realism. We observe that Diffusion-based synthesis produces the most realistic and distributionally aligned fall data. To further evaluate the impact of synthetic data, we train a long short-term memory (LSTM) model offline and test it in real time using the SmartFall App. Incorporating Diffusion-based synthetic data improves the offline F1-score by 7–10% and boosts real-time fall detection performance by 24%, confirming its value in enhancing model robustness and applicability in real-world settings.

## 1. Introduction

Falls are a leading cause of injury and one of the primary contributors to unintentional deaths among adults aged 65 and older worldwide [1,2]. This high incidence highlights the urgent need for effective prevention and intervention strategies to protect the health and well-being of the elderly population. In response, research on fall detection technologies has grown substantially, particularly in the use of wearable devices such as smartwatches and inertial measurement unit (IMU) sensors. These devices are increasingly favored due to their portability, affordability, and non-intrusive nature [3].

There is also an increasing interest in leveraging deep learning models for learning the dynamical behavior of falls and the accurate detection of fall events using a wearable device [4]. However, in many physiological processes like the onset of falls, the dynamics are exceedingly complex, and deep learning techniques are not effective, especially when given a limited amount of data for training. Moreover, a fall is a rare event, and it is impossible to collect a large amount of fall data. In the past, researchers have embarked on collecting simulated fall data from young adults in a controlled environment. The data collection process is costly and laborious. In the context of our SmartFall multi-modal (SmartFallMM) dataset (the work is under review), it took a whole month of effort to collect data from 40 people. This excludes the effort needed to implement suitable data collection systems for various types of IMU sensors, as well as the effort in recruiting the participants. Therefore, integrating deep learning techniques into these systems faces a significant challenge: the severe shortage of real human fall data for training. To address this, synthetic data is increasingly being used [5,6] to overcome this limitation. Data augmentation techniques are a widely recognized method for mitigating the problem of small datasets. By artificially expanding the available data through various transformations and modifications, these techniques help improve the robustness and generalizability of deep learning models.

Recently, generative AI has gained prominence in research, particularly in the field of synthetic data generation across various domains, including images and time-series data. Techniques such as Generative Adversarial Networks (GANs) [7,8,9], Variational Autoencoders (VAEs) [10], and Diffusion models [11,12] are increasingly utilized for creating synthetic time-series data. These methods are capable of generating data that closely resembles real-world data by learning complex patterns and structures from existing data. In particular, the Diffusion-based generative AI model has emerged recently as a popular tool for data generation, demonstrating exceptional performance across a variety of applications.

Platforms like YouTube with video datasets of people’s daily life activities, including free fall (i.e., the downward motion phase during a fall when the body experiences minimal support and acceleration approaches gravity [13]) can be another source for obtaining authentic fall data. Unlike the majority of currently available fall datasets, which rely on simulated falls, leveraging video content from public video sources could provide a more accurate representation of real-world falls, leading to better training data for fall detection systems. Pose estimation has become a cornerstone in analyzing human motion across various industries, including sports and healthcare [14]. By harnessing machine learning and computer vision, these techniques can extract skeletal data from videos, offering detailed insights into body movement. Using pose estimation techniques on video content from publicly accessible video sources, researchers might be able to create realistic fall data that closely mirrors actual human movements.

However, there is no comparative study yet on the impact of synthetic data on the performance of fall detection models trained with a mix of real and synthetic fall data, particularly within real-world applications. In general, researchers tend to evaluate the quality of generated data using similarity metrics such as principal component analysis (PCA) and t-distributed stochastic neighbor embedding (t-SNE) [15], which capture distributional resemblance but not necessarily functional performance. Therefore, to validate both the practical utility and quality of the generated data, it is crucial to demonstrate that models trained with synthetic data can achieve high F1-scores in real-world scenarios.

To address the persistent challenge of limited real-world fall data, this paper introduces a novel hybrid framework that combines three complementary approaches: traditional augmentation techniques, Diffusion-based generative modeling, and video-based pose estimation. This paper presents an experimental study of the effectiveness of these techniques for generating multivariate synthetic fall data. Additionally, it explores different metrics to evaluate the quality of generated synthetic data. We examine and compare several methods for generating and augmenting fall data, including three established time-series data augmentation techniques, such as jittering, magnitude warping, and rotation, a Diffusion-based generative AI technique, as well as a novel approach involving pose estimation from video recordings of falls in older adults.

The main contributions of this paper are as follows:

- The implementation of two distinct synthetic data generation techniques for time-series data, including video-based extraction (pose estimation) and generative AI-based (Diffusion) generation, where we used traditional data augmentation methods as a baseline for performance comparison.
- A comparative evaluation of fall detection model performance, incorporating both real and synthetic data across three different public fall datasets using three different synthetic data generation techniques.
- A demonstration that synthetic data produced using the Diffusion approach and pose estimation from video recordings can enhance the fall detection model’s performance in real-world scenarios using the SmartFall App.
- An exploration of various quantitative evaluation metrics provides insights into the quality and utility of synthetic data for effective model training.

The remainder of this paper is structured into five main sections. Section 2 reviews recent advancements in synthetic data generation, including the use of generative AI techniques such as GANs, Diffusion models, and pose estimation, as well as general data augmentation methods. Section 3 details the overall methodology, covering the synthetic data generation processes, the datasets employed, the deep learning model used for fall detection, and an explanation of the experimental setup. Section 4 outlines three synthetic fall data generation methods and compares their suitability for fall detection. Section 5 presents the results of both offline and real-time evaluations. Finally, Section 6 offers conclusions, and Section 7 discusses potential directions for future research. This paper is an extended version of the short paper presented at AIME 2024 [16].

## 2. Related Work

The generation of synthetic time-series data for accelerometer-based fall detection systems has been a significant area of research. Various generative techniques have been proposed to address this challenge, each with its strengths and limitations. In this section, we discuss the techniques that fall within the scope of this study.

### 2.1. Generative Adversarial Networks (GANs)

Generative Adversarial Networks (GANs) have been widely used for synthetic time-series data generation. For example, TimeGAN [7,9] integrates the strengths of both GANs and Recurrent Neural Networks (RNNs) to capture temporal dependencies in data. Zhang et al. [17] also applied GANs to learn the conditional probability distribution of essential features in the real dataset and generate samples based on the learned distribution. Other frameworks, like TSGM [10] with GANs and Variational Autoencoders (VAEs) and DoppelGANger [18] with GANs only, offered flexibility for generating synthetic data with complex dependencies.

In the medical domain also, GANs have been employed to generate synthetic medical health records, which are used to train machine learning models for disease diagnosis and treatment prediction. For example, the paper [8] showcases the use of GANs to create synthetic medical records that maintain the temporal and sequential patterns found in real datasets. This technique addresses significant challenges, such as patient privacy concerns and the scarcity of large, diverse datasets for training machine learning models. Another method, MedGAN [19], used an autoencoder and GANs to generate synthetic patient records close to the real data of patients.

However, GANs are known to suffer from instability during training and mode collapse, where the generator produces limited diversity in outputs. Also, GAN-based models often require large amounts of labeled data and extensive computational resources, which are not always available for fall detection datasets. These issues can be particularly problematic when aiming to generate a variety of scenarios necessary for effective fall detection systems. The complexity and extensive tuning required for GANs can be a hindrance, especially when dealing with the high-dimensional and noisy nature of accelerometer data.

### 2.2. Diffusion Models

Diffusion models have recently emerged as a powerful tool for generating synthetic time-series data. These models effectively handle complex data distributions in domains like healthcare and finance because they can capture intricate patterns and dependencies within the data. For example, the TS-Diffusion model [11] effectively generates high-fidelity and diverse time-series data by leveraging Neural Ordinary Differential Equations (ODEs). Similarly, Diffusion-TS [20] introduces interpretability by incorporating Fourier-based loss terms, allowing it to uncover time-series components like seasonality and trends. Tian et al. [12] proposed a method that used Denoising Diffusion Probabilistic Models (DDPM) to generate diverse and realistic synthetic time-series data of Electronic Health Records (EHRs).

Despite their strengths, Diffusion-based methods face challenges in capturing subtle and rapid motion patterns critical for accelerometer-based fall detection. For example, TS-Diffusion struggles with representing short, transient events, potentially overlooking key biomechanical details. Although Diffusion-TS enhances interpretability through frequency-domain analysis, it often fails to reconstruct abrupt, non-stationary movements typical of falls. In contrast, DDPMs show promise by effectively modeling small datasets and dynamic temporal structures, making them better suited for realistic fall data generation.

### 2.3. Pose Estimation Techniques

Pose estimation methods have enabled the analysis of human motion in various fields, such as sports, healthcare, and animation. These methods are extensively utilized to extract kinematics or skeleton data from videos by leveraging advancements in computer vision and machine learning to track human body joints and generate detailed motion data. For instance, recent work [14] has focused on improving 2D human pose estimation across rare camera views using synthetic data, particularly in sports applications, by incorporating diverse datasets that include video footage from platforms like YouTube.

Despite the widespread application of pose estimation for capturing complex motion data, there remains a significant gap in datasets specifically targeting fall detection scenarios. While pose estimation is often used to extract skeleton data, no study has demonstrated converting this data into accelerometer data for fall detection. Addressing this gap requires collecting and generating fall-specific data, using pose estimation to ensure the data reflects real-world fall events involving the wrist joints, used in an accelerometer format to enhance the training of fall detection systems.

### 2.4. Augmentation Techniques

Various augmentation techniques have been employed to generate synthetic time-series data to enhance the training and performance of machine learning models [21]. For example, Dynamic Time Warping (DTW) [22] allows for the creation of new time series by averaging existing series with varying weights. Other techniques like window warping and suboptimal warping [23] manipulate the time-series data to simulate different temporal patterns. Jittering, scaling, permutation, and frequency-based augmentations [24] are also commonly used to introduce variability and simulate real-world noise and conditions.

Despite the advantages of augmentation techniques, generating synthetic data, which accurately captures the variety and complexity of fall scenarios, remains challenging. Falls can vary greatly in direction, speed, and impact, and it can be difficult for standard augmentation techniques to simulate these nuances effectively. Additionally, there is a lack of studies demonstrating the reliability of these techniques specifically for fall data, which highlights the importance of evaluating their impact on machine learning models. Conducting further research into these techniques is crucial for validating their effectiveness and enhancing the robustness of fall detection systems.

## 3. Methodology

**Figure 1:**
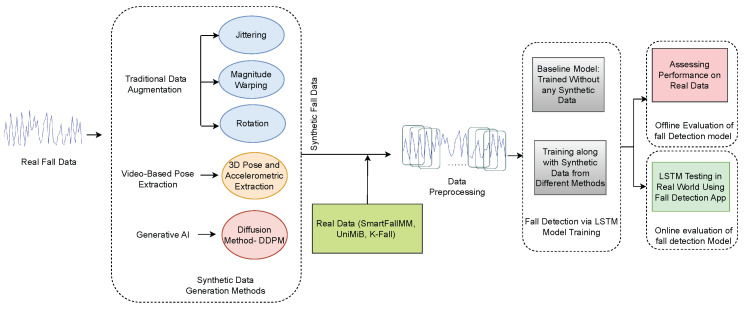
Schematic representation of the overall approach.

Figure 1 illustrates the complete workflow of the proposed methodology. A detailed explanation of each component in the workflow is provided below.

### 3.1. Datasets

We utilized three publicly available fall-related datasets for various synthetic data generation techniques, along with a video dataset and supplementary fall recordings sourced from YouTube. The public datasets are SmartFallMM (https://github.com/txst-cs-smartfall/SmartFallMM-Dataset) (accessed on 29 May 2025) data collected in our laboratory, the UniMiB [25], and the K-Fall [26]. These datasets consist of various simulated falls and daily activities carried out by healthy young adults. The video dataset [27] includes recordings of older adults falling in a long-term care facility but with limited availability. To supplement this, we scraped YouTube for “falls of elderly people” videos. It is worth pointing out that the YouTube-sourced videos were not used as a benchmark dataset but rather as a supplementary source to enhance training diversity. Only videos with clearly observable fall dynamics were used to ensure data validity.

The UniMib dataset was collected to enhance human activity recognition and fall detection using data from wearable devices, such as smartphones. This dataset was collected using a Samsung Galaxy Nexus I9250 smartphone, equipped with a Bosch BMA220 triaxial low-g acceleration sensor. Additionally, the smartphone’s built-in microphone recorded audio signals at 8000 Hz, aiding in data annotation. During data collection, participants placed the smartphone in their front trouser pockets, alternating between the left and right sides. Acceleration and audio data were recorded using a custom mobile application and stored in separate files on the smartphone. For data annotation, claps were performed by an external subject before and after each activity or fall to guide the participant and to minimize movement artifacts that could interfere with the recorded sensor data. A 3 s signal window centered around each peak was used for training. The sampling rate of this dataset is 50Hz. The data was collected from 30 participants, aged between 18 and 60. It covers a wide range of activities, including 9 types of daily life activities (ADLs) and 8 different types of falls.

The K-Fall [26] dataset was collected for detecting pre-fall, fall, and post-fall events. It includes data from 32 healthy young adults who performed 21 daily activities and 15 simulated falls, with each activity repeated 5 times. Data collection utilized a custom-designed system that synchronizes sensor data with high-frequency video. A nine-axis inertial sensor was attached to the lower back of participants, recording data from a three-axis accelerometer, gyroscope, and magnetometer at a frequency of 100 Hz. This sensor also provided orientation measurements in Euler angles with data integrated and filtered using an extended Kalman filter. The data was transmitted via Bluetooth to a Raspberry Pi 4, which managed the recording process using a custom graphical user interface (GUI) and handled synchronization between the sensor and a Raspberry Pi HQ camera. The camera captured video at 90 frames per second to provide synchronized visual data. The video recordings have been primarily used for accurate labeling of fall events. For this study, only the accelerometer data was used.

SmartFallMM is a multi-modal dataset collected in our laboratory, involving both older and young adults. The dataset includes data from 24 older adults between the age of 60 and 93 and 16 young adults aged between 20 and 30. For this paper, we utilized data from 12 young adult participants (7 male and 5 female). At the time of this experimental study, there were only 12 younger people data that were labeled. Participants were instructed to simulate five types of falls (front, back, left, right, and rotational) on an air mattress and perform nine types of ADLs, with five repetitions each. Three Azure Kinect cameras recorded all falls and ADLs, capturing the skeletal posture associated with each activity for cross-modal learning research and accurately labeling the beginning and ending of all trials. The Meta Sensor (from MBIENT lab) was positioned on the right wrist, the Huawei watch on the left wrist, the Nexus smartphone on the right hip, and another Meta Sensor on the left hip. This setup allowed us to collect data from four crucial joints of the human body during various movements. This data collection process was approved by the Institutional Review Board (IRB 9461) at Texas State University. Only the accelerometer data from the Huawei Smartwatch was used to generate synthetic fall data.

The final dataset we used includes a collection of videos [27] featuring older adults experiencing falls in long-term care facilities, as well as short clips of falls of older adults from YouTube. The YouTube videos capture candid fall scenarios, providing an alternative, realistic representation of falls in everyday environments. We used 24 videos from YouTube in this study and 34 videos from the two long-term care facilities. These falls occurred exclusively in shared spaces like lounges, dining rooms, and hallways within the facilities.

For the SmartFallMM and UniMiBand K-Fall public fall datasets, all the existing fall samples in the datasets were utilized for synthetic data generation. In the video extraction method, we utilized all available fall videos. The original real fall datasets were then expanded by progressively adding synthetic fall data until a balance was achieved between fall and no-fall instances. This strategy ensures even distribution within the datasets, enhancing the reliability of the subsequent analysis.

### 3.2. Fall Detection Learning Model

**Figure 2:**
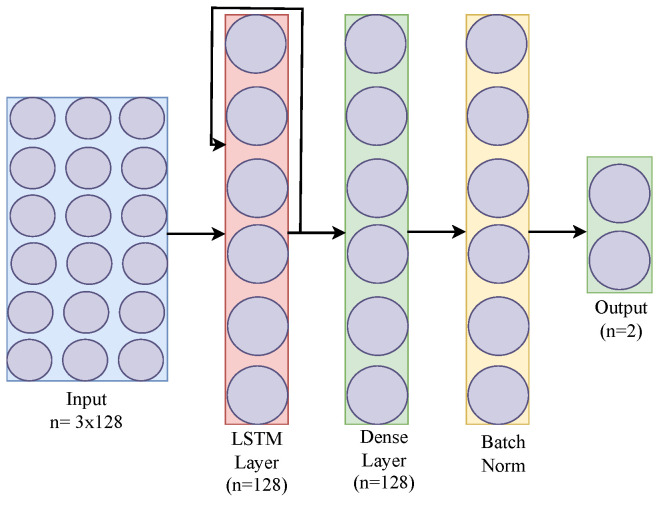
Basic LSTM architecture.

We employed a simple long short-term memory (LSTM) deep learning model, which has been shown to be effective for time-series data due to its capability to learn temporal dynamics. Our LSTM model’s input layer has 3 nodes corresponding to the accelerometer’s x, y, and z vectors, with an input shape of (W, 3, 64), where W represents the window size set to 128, capturing around four seconds of data, and 64 is the batch size. The training process utilizes the Binary Cross Entropy (BCE) loss function with the ADAM optimizer. This model has been successfully deployed and tested in our SmartFall App [28], where it demonstrated better performance compared to the 1D CNN, Gradient Boosting, and Random Forest methods [29]. Figure 2 represents the basic LSTM architecture.

This LSTM model consists of four layers, including an LSTM layer, a Batch Normalization layer, and one dense layer with the number of neurons matching the window-size parameter.

### 3.3. Data Preprocessing, Training, and Evaluation

In the data preprocessing phase, we utilized the sliding window technique to divide the dataset into overlapping windows, applying a step size of 10, which introduces ten new data points into each new window. For consistency across all experiments, the window size was set to 128. This size was chosen based on prior research [28], which demonstrated that it effectively captures the necessary fall patterns for LSTM models. Our experiments focused exclusively on the basic LSTM model, which was tested under various training scenarios. Baseline models were trained using only the original datasets, i.e., without the addition of any synthetic data. The dataset was split into training, validation, and test sets in a 70/20/10 ratio. A 5-fold validation approach was implemented, with each fold consisting of data from 20% of individuals for validation, 10% for testing, and 70% for training. These baseline models provided a reference for assessing the impact of synthetic data.

To evaluate the effectiveness of synthetic data, we trained new LSTM models using a combination of the original training dataset and synthetic data generated by the various methods described in Section 4. Across all three datasets, SmartFallMM, UniMiB, and K-Fall, we generated and included synthetic data only for the fall class, without synthesizing non-fall or activities of daily living (ADL) data. This decision was driven by the fact that real-world data already contains a substantially larger amount of non-fall samples compared to fall events. To prevent class imbalance, we carefully determined the number of synthetic fall samples added to the training data for each dataset, ensuring that the final training set maintained a balanced or nearly balanced distribution between fall and non-fall events (approximately a 50/50 ratio). Validation and testing were performed exclusively on real data to fairly assess the model’s generalization performance.

To address potential concerns about preserving extreme variations in fall dynamics, we ensured that no statistical outlier removal or normalization was applied to the original fall data used for training any of the synthetic data generation methods. This design choice was intentional to retain exceptionally high kinetic and kinematic patterns, such as abrupt accelerations or sharp impacts, that often characterize real-world fall events.

The experimental results are presented in the Results Section. The effectiveness of the synthetic data was evaluated using standard metrics such as precision, recall, F1-score, and accuracy to determine which method for generating synthetic data was most effective in addressing the lack of real fall data.

We also tested the most effective model trained with the addition of synthetic data in a real-world environment using the SmartFall App. This real-time assessment involved three student participants, with approval from IRB 9461 at Texas State University. Each participant wore a smartwatch running the SmartFall App, simulated five different types of falls on a queen-size air mattress, and carried out a specified list of seven activities of daily living (ADLs). Both true positive and false positive predictions were documented during the evaluation. In our real-world testing of the fall detection app, the participants performed five repetitions of each of five different fall types, totaling fifteen falls, alongside seven daily life activities to assess false alarm rates. For the LSTM model trained with SmartFallMM data combined with Diffusion-based synthetic data (SF w DF), the system achieved high recall (0.97), missing none or at most one fall, and produced approximately four false positive alerts during daily activities. Similarly, for the model incorporating video-extracted synthetic data (SF w VE), recall was slightly lower (0.95), with around one missed fall and about five false positives. These results demonstrate strong sensitivity in detecting falls, though further work is needed to reduce false alarms and improve practical deployment.

## 4. Synthetic Data Generation

This section presents the three distinct strategies we employed to generate synthetic fall data: traditional data augmentation techniques, video-based extraction through pose estimation, and Diffusion-based generative modeling. Table 1 summarizes the key characteristics, strengths, and constraints of each of these synthetic data generation techniques, providing insight into their suitability for fall detection applications.

### 4.1. Basic Data Augmentations

Data augmentation has become a widely adopted technique in computer vision, but its application in time-series analysis is still relatively underexplored. In our study, we implemented three specific data augmentation methods: jittering [30], magnitude warping [30], and rotation [21].

Jittering involves adding random Gaussian noise to time-series data. By introducing small, random perturbations, jittering helps simulate real-world variations and imperfections that may not be present in the original dataset. This augmentation makes the model more robust to noisy data, improving its ability to generalize to new, unseen data while preserving the core patterns in the time series.

Magnitude warping adjusts the amplitude of the time-series data by applying a scaling factor derived from a cubic spline curve. The cubic spline curve modifies the magnitude of the entire time series in a smooth and controlled manner. This technique allows for the exploration of different signal strengths and ranges, which can be crucial for models that need to perform well across various magnitudes of data, effectively helping the model learn to handle amplitude variations.

Rotation simulates different sensor placements by applying rotations to the time-series data. For example, data recorded from a sensor on the left wrist might be rotated to mimic the sensor being placed on the right wrist. This augmentation introduces variability in sensor orientation without changing the actual labels associated with the data. It helps the model learn to recognize patterns despite differences in how data is collected, making the model more adaptable to different sensor configurations and improving its generalization to diverse scenarios.

### 4.2. Diffusion Method—DDPM

Denoising Diffusion Probabilistic Models (DDPMs) [15,31] represent a class of generative AI models that have demonstrated remarkable success in synthesizing high-quality data across domains, such as images and audio.

**Figure 3:**
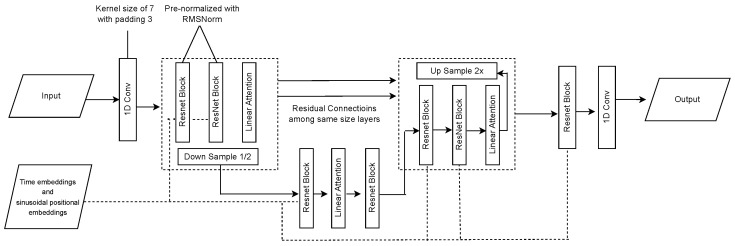
Schematic illustration of the U-Net architecture adapted for time-series data in the Diffusion model.

We have integrated a Diffusion model with the U-Net architecture adapted from previous work [15]. Initially developed for image analysis, the U-Net architecture, as illustrated in Figure 3, has been adapted for time-series data by employing one-dimensional (1D) convolutional layers with a kernel size of 7 and padding of 3, enabling it to capture crucial temporal dependencies in the data.

The time-series input is first processed with RMSNorm to ensure stable training. The network architecture, comprising ResNet blocks and Linear Attention units, executes downsampling and upsampling operations to refine features and preserve temporal information. Each block incorporates time and sinusoidal positional embeddings, enabling the model to effectively respond to Diffusion timesteps and sequence positions.

Li’s original model [15] faced difficulties in effectively handling accelerometer data with varying lengths across different fall scenarios, which made it challenging to standardize the input for the model. Additionally, the model lacked a reliable normalization method, which could lead to inconsistencies during training. To address these issues, we introduced a padding strategy during the preprocessing stage. Specifically, we padded each sample with its last value, which allowed us to standardize the input size across all data points without discarding any important information. This modification not only stabilized the model’s performance but also ensured that no critical details were lost during data preparation. Equations (1) and (2) detail the strategies we used.

Adjusting Trial Lengths:

(1) $$L_{\mathrm{new}}=\left\lceil \frac{L_{\max}}{8}\right\rceil \times 8$$

Padding Operation:

(2) $$x^{\prime }=\mathrm{pad}\left(x,L_{\mathrm{new}}-L_{\mathrm{current}},v\right)$$

where

- *x* is the original trial data;
- $L_{\max}$, $L_{\mathrm{current}}$, and $L_{\mathrm{new}}$ are the max, current, and new lengths, respectively;
- pad denotes the padding operation;
- *v* is the value used for padding, the last element of *x*.

To address normalization challenges, particularly in training stability with deep networks, we introduced RMSNorm [31] within the ResNet blocks of the U-Net structure. This normalization technique contributes to stable training dynamics. The use of scaling activation magnitudes improves convergence speed and model performance with variable-length time-series data. Implementing padding strategies and RMSNorm ensures consistent handling of variable-length data, optimizing the generation of synthetic time-series data and enhancing fall detection models.

### 4.3. Extraction of Fall Data from Video via Pose Estimation

In 3D pose estimation from video, deep learning methods, particularly neural networks, are widely used. Researchers utilize large datasets, including real-world data, to improve how well models perform across different situations. A common approach is using 2D-to-3D estimation techniques, which rely on 2D keypoints to make computations more efficient and allow for real-time results. There is also a trend toward end-to-end systems that directly predict 3D poses, making the process more straightforward. We have implemented the methodology outlined in [32,33]. To accurately extract fall data, we processed 34 publicly available videos from [27] and 24 additional YouTube videos. Our method involved cropping the video frames to isolate the falling person, focusing on reducing extraction time and highlighting relevant data. We retained 1 to 2 s of footage before and after the fall. Each video was adjusted for resolution and brightness as needed.

Using 3D pose estimation, we extracted the positions of 17 joints from the human skeleton in each video. For synthetic data generation, we focused on specific joint positions depending on the target dataset. For instance, to match the SmartFallMM dataset, we extracted accelerometer data from the left wrist joint. For the UniMiB dataset, we extracted data from both the left and right hips. Acceleration data was derived from 3D keypoints by calculating the velocity from position changes and then the acceleration from velocity changes. To simulate accelerometer signals more precisely, we computed joint-specific acceleration data from the extracted 3D coordinates by calculating discrete second-order differences across consecutive frames. For example, considering the left wrist joint, let p(f)=[x(f),y(f),z(f)] denote the 3D positional coordinates at frame *f*. The acceleration vector a(f) is estimated using the discrete second derivative:

(3) $$a\left(f\right)=\frac{p(f+1)-p(f)}{\Delta t^{2}}$$

where Δt is the time interval between video frames (Δt=1/46s). Each axis component, $a_{x}\left(f\right)$, $a_{y}\left(f\right)$, and $a_{z}\left(f\right)$, was computed independently and scaled to produce acceleration values in ${m/s}^{2}$.

**Figure 4:**
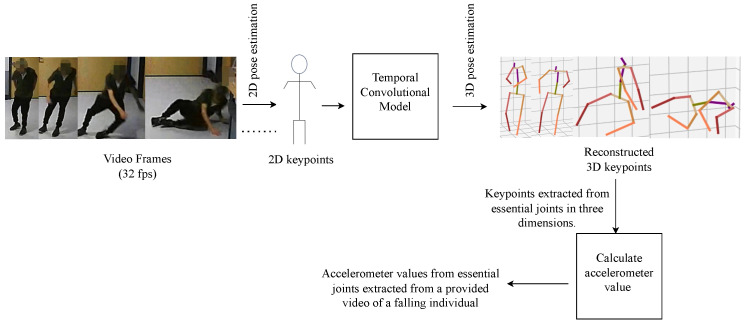
Pipeline for extracting synthetic accelerometer data from video frames. Colored segments in the 3D skeletons represent different body parts (e.g., arms, legs, torso), with distinct colors used to aid visual interpretation. The dots indicate the presence of additional frames in the video sequence.

Figure 4 illustrates the methodology for obtaining the accelerometer readings from the joint positions in the video. This approach yielded approximately 54 fall samples from the videos. To ensure data quality, we addressed occlusions by discarding samples where key joints (e.g., the left wrist or hips) were not reliably tracked due to motion blur, low resolution, or brief occlusion during the fall. Additionally, we applied temporal smoothing to correct minor discontinuities in joint trajectories. Only sequences with stable and continuous joint tracking across the fall window were retained for synthetic accelerometer extraction.

### 4.4. Evaluation Metrics for Synthetic Data Quality

To evaluate the quality of the synthetic time-series data, we used the following metrics:

- **FID Score**: Measures the difference in contextual representations of synthetic and real samples, based on the Fréchet distance between feature distributions. The score ranges from 0 to *∞*, where lower values indicate higher similarity and thus better quality [34].
- **Discriminative Score**: Evaluates how well a binary classifier can distinguish real from synthetic data. Typically expressed as an accuracy percentage ranging from 50% to 100%. A lower score, close to 50%, indicates higher similarity between real and synthetic data, as it implies the classifier cannot reliably differentiate them [9].
- **Predictive Score**: Measures the utility of synthetic data for forecasting future time steps. It is often reported as a prediction error metric (e.g., RMSE) or as accuracy for classification tasks. The score ranges from 0 to *∞* (for error metrics), with lower values indicating better predictive utility [9].
- **Jensen–Shannon Divergence (JSD)**: Quantifies the similarity between the probability distributions of real and synthetic data. JSD values range from 0 to 1, where 0 indicates identical distributions, and higher values indicate greater divergence [35].
- **Kolmogorov–Smirnov (KS) Test:** A non-parametric statistical test used to compare two samples and determine whether they come from the same distribution. Unlike normality tests that only assess fit to a specific distribution, the K–S test directly compares empirical distributions, making it especially useful for evaluating model outputs or validating synthetic data against real samples [36]. The test outputs a D-statistic ranging from 0 to 1, where lower values suggest greater similarity between distributions. A corresponding p-value indicates statistical significance; higher p-values imply no significant difference between the distributions.

## 5. Results

For consistency and clarity throughout the Results Section, we use the following abbreviations for datasets and augmentation methods: SF refers to SmartFallMM, UM denotes UniMiB, and KF represents K-Fall. Regarding the synthetic data generation and augmentation methods, Jit stands for jittering, VE denotes video extraction, MW represents magnitude warping, and Ro corresponds to rotation. These abbreviations will be used consistently in all the result tables and discussions. Additionally, to ensure completeness and benchmark against state-of-the-art methods, we include a comparison with Diffusion-TS, which is a recent generative AI-based approach.

### 5.1. Quality Evaluation Results for Synthetic Data

Table 2 presents a comprehensive comparison of the evaluation metrics for the three datasets across multiple methods, the Context-FID Score, Discriminative Score, Predictive Score, Jensen–Shannon Divergence (JSD), and Kolmogorov–Smirnov statistic (KS), where lower values indicate better performance. The bolded values in the table indicate the best performance for each score. The proposed Diffusion (ours) model achieves the lowest Context-FID scores (e.g., 0.409 on SmartFallMM and 1.523 on UniMiB), demonstrating better contextual fidelity. The model achieves notably low discriminative scores (e.g., 0.704 on SmartFallMM and 0.500 on K-Fall), substantially outperforming Jit (0.833 and 0.921) and MW (0.801 and 0.879). This suggests that the generated data more accurately captures the statistical and temporal dynamics of real accelerometer signals. Moreover, the model achieves the lowest JSD values across all the datasets, 0.056 on SmartFallMM, 0.009 on UniMiB, and 1.610 on K-Fall, indicating closer distributional alignment with real data than other approaches, such as Diffusion-TS (0.241, 0.874, and 0.458, respectively) and Jit (0.295, 0.981, and 0.598). This alignment suggests a better preservation of the underlying statistical properties essential for realistic synthetic data.

For KS, across all three datasets, our proposed Diffusion-based generator consistently achieves the smallest average $\bar{D}$: 0.142 on SmartFallMM and 0.095 on UniMiB, indicating that its synthetic accelerometer data most closely match the real distributions. On the K-Fall dataset, the video-extracted method performs slightly better than proposed Diffusion model ($\bar{D}=0.321$ vs. $\bar{D}=0.345$). Jit (0.314–0.434), MW (0.211–0.377), and Ro (0.188–0.459) yield substantially higher $\bar{D}$ values, underscoring their inability to capture true sensor statistics. Notably, UniMiB proves easiest to model (all methods $\bar{D}<0.13$), whereas K-Fall remains the hardest, with even the best method exceeding $\bar{D}=0.32$. These results demonstrate that learned generative approaches, particularly our Diffusion variant, offer better distributional fidelity compared to traditional augmentations, especially on complex real-world time-series signals. In summary, the proposed Diffusion (ours) model consistently outperforms all the competing methods across all the datasets and metrics, highlighting the robustness and generalizability in generating better-quality, real-like, and task-relevant synthetic data for fall detection applications.

**Figure 5:**
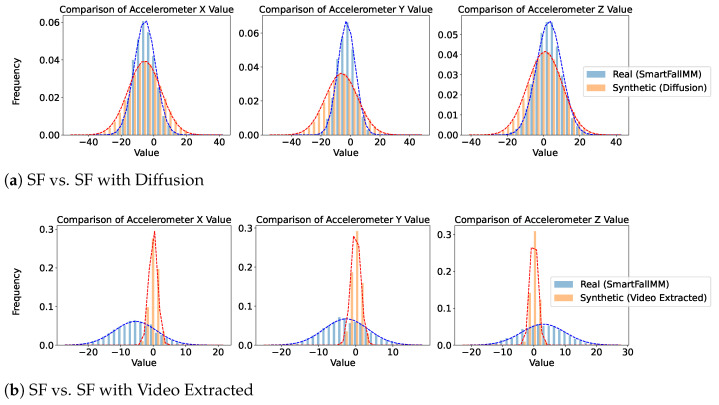
Comparison of real and generated data for SmartFallMM. Blue represents the real data distribution, while orange denotes the synthetic data distribution.

**Figure 6:**
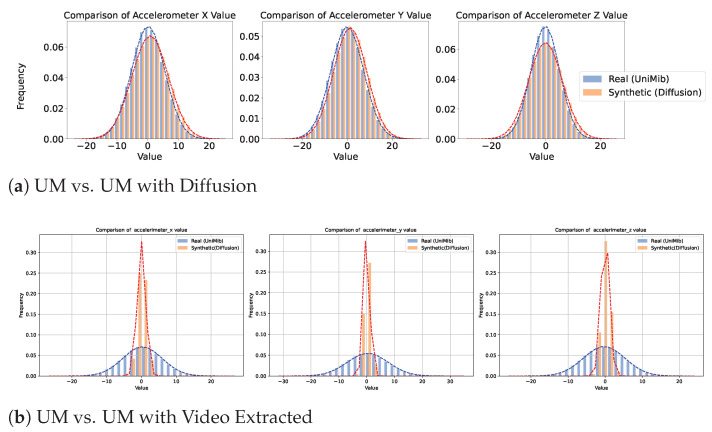
Comparison of real and generated data for UniMiB. Blue represents the real data distribution, while orange denotes the synthetic data distribution.

**Figure 7:**
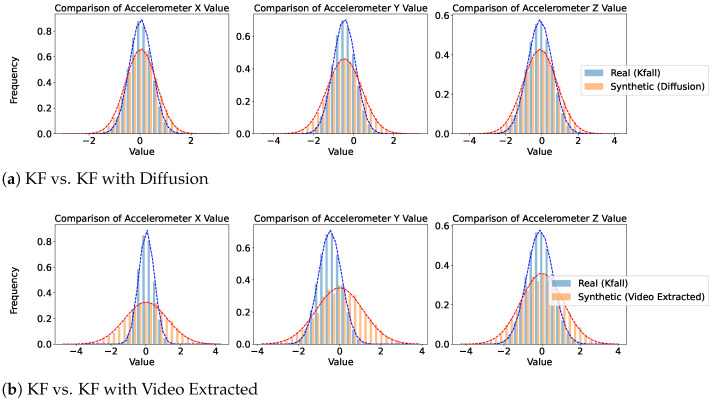
Comparison of real and generated data for K-Fall. Blue represents the real data distribution, while orange denotes the synthetic data distribution.

Figure 5, Figure 6 and Figure 7 provide a comparative analysis of real and synthetic fall data, focusing on Diffusion-generated and video-extracted synthetic data, the two best-performing generation methods, across the three datasets. Each dataset comprises two comparison scenarios: “Real vs. Synthetic (Diffusion)” and “Real vs. Synthetic (Video Extracted).”

We analyzed the statistical distribution of the real and synthetic data to assess their similarity. In all the plots shown in this figure, the x-axis represents accelerometer values collected from wearable devices such as smartwatches, depending on the specific dataset. These values measure acceleration along different axes (X, Y, and Z) and are typically expressed in meters per second squared (m/s^2^). Higher absolute values indicate stronger movements or impacts detected by the wearable sensor. The y-axis indicates the frequency of these values. These plots illustrate how well the synthetic data reflects the statistical distribution of real data, highlighting the similarity in their normal distributions. In the SmartFallMM, UniMiB, and K-Fall datasets, the Diffusion-generated synthetic fall data closely match the distribution of the real fall data, indicating a strong alignment. Notably, the synthetic data derived from YouTube videos via video extraction also demonstrated a similar distributional pattern to those in the benchmark datasets, supporting the validity of these videos as a viable supplementary data source. While the video-extracted synthetic data exhibits a narrower distribution range, particularly in the accelerometer values along the x, y, and z axes, compared to the real fall data, this indicates that the synthetic data captures a more focused spectrum of fall dynamics. This focused representation can be useful for emphasizing key fall characteristics while still providing valuable insights into the dynamics of falls. Overall, these results suggest that the synthetic data effectively replicates the statistical variability observed in real falls across all three datasets.

### 5.2. Offline Evaluation of Fall Detection Model

Table 3 presents an overview of the comparative evaluation performed on the three datasets, incorporating the synthetic data generated using three different methods. The values highlighted in bold and italics correspond to the highest and second-highest improvements, respectively, observed in the fall detection task. In this analysis, we present the average results from 5-fold validation, which involves conducting five experiments for each dataset, both with and without synthetic data. The average precision, recall, F1-score, and accuracy from these experiments are reported for each dataset. To enhance clarity, we refer to SmartFallMM as SF, UniMiB as UM, K-Fall as KF, video extraction from older home people along with YouTube videos as VE, jittering as Jit, magnitude warping as MW, and rotation as Ro. The results obtained by solely using the SmartFallMM dataset (SF) without any data augmentation yield an F1-score of 0.72 and an accuracy of 0.77. After applying synthetic data generated from various techniques, the most promising result obtained for SF is by incorporating the combination of the VE method, achieving an F1-score of 0.84, which indicates an improvement of almost 12%.

Using Diffusion-generated data also showed improved performance with an F1-score of 0.80 than the baseline result. These results demonstrate that Diffusion and video extraction-based methods are both effective in synthesizing accelerometer data for fall detection. They enhance the diversity of our dataset by covering a broader range of fall scenarios. We also evaluated and compared our findings using two other public datasets: UniMiB and K-Fall. Both K-Fall and UniMiB exhibited performance improvements when utilizing Diffusion-generated data and the video extraction method. Specifically, UniMiB showed an approximate 8% improvement, while K-Fall demonstrated a 12% increase compared to the baseline results. Diffusion-generated data demonstrated improvements across all three datasets. Also, when additional video data was incorporated (YouTube-scraped video) using the video extraction method, the results improved compared to using only the video dataset from the long-term facility presented in our previous work [16]. This suggests a promising approach by including more videos of elderly individuals from sources like YouTube to extract skeleton data and augment real-world fall data.

As observed, the model trained with synthetic data from the proposed Diffusion method consistently outperforms the baseline across all the datasets. Compared to the baseline, the proposed method improves the F1-score by 11.11% on SF (from 0.72 to 0.80), 13.51% on UM (from 0.74 to 0.84), and 16.22% on KF (from 0.74 to 0.86). Similarly, the accuracy increases by 6.49% on SF (from 0.77 to 0.82), 7.89% on UM (from 0.76 to 0.82), and 10% on KF (from 0.80 to 0.88). These consistent and substantial gains reinforce the effectiveness of the proposed Diffusion method in enhancing fall detection performance. While the video-extracted (VE) method also performs competitively (e.g., highest F1-score on SF and UM), the proposed Diffusion method demonstrates a more balanced and consistently high performance across all the metrics and datasets. Based on these results, we recognize both approaches as effective for improving fall detection performance in our framework.

These offline-evaluation results not only validate the effectiveness of our methods but also emphasize the role of offline evaluation in guiding real-world deployment strategies. In fall detection, where real-world fall data is both rare and ethically difficult to obtain, consistent improvements across multiple datasets suggest that the model is learning robust, transferable patterns. These findings establish a foundation for real-time deployment and guide further validation efforts in operational settings.

### 5.3. Real-Time Evaluation of Fall Detection Model

Table 4 showcases the real-world evaluation results for the top-performing offline model. Real-world testing was conducted with the SmartFall App, which is designed to operate with data sensed from a wearable watch. The presented values represent average performance across three participants, with the bold values showing the highest gain. Initially, we evaluated the SmartFall App using a basic LSTM model trained solely on real SmartFallMM data (SF), which achieved an F1-score of 0.62. We then evaluated the top models trained with combinations of synthetic and real data, following the protocol described in Section 3.3. These include models trained using Diffusion-generated data (SF w DF) and video-extracted data (SF w VE).

The best real-world performance was observed from the model trained with Diffusion-generated data, achieving an F1-score of 0.86, which is an improvement of approximately 24%. The model trained with VE data also performed well, yielding an F1-score of 0.82 (a 20% improvement). These results closely mirror the trends observed in our offline evaluation. Specifically, the model trained with Diffusion-generated data showed an F1-score improvement of 11.11% offline (from 0.72 to 0.80) and 24% in real-world testing (from 0.62 to 0.86). Similarly, the video extraction method yielded an offline F1-score gain of 19.44% and a corresponding 20% boost in real-time performance. This strong correlation between offline and online results supports the practical utility of offline evaluation as a reliable indicator of real-world effectiveness, especially when robust synthetic data generation techniques are applied.

### 5.4. Limitations

Despite the promising results, this study has a few limitations. First, the current model does not account for individual-specific movement patterns, which may affect generalizability across diverse populations. This highlights the need for personalization techniques that tailor the model to each user’s activity profile. Second, our data pipeline relies solely on accelerometer input, omitting additional sensor modalities such as gyroscope or barometric data that could enhance contextual understanding and improve fall detection accuracy. Third, the video extraction process, though effective, depends on manual frame selection, which is time-consuming and may introduce human bias. This limits scalability and consistency, calling for automated frame selection strategies. Finally, the real-world testing reported in Table 4 was conducted on a limited sample of only three younger participants and involved simulated falls. While this provides a proof of concept for evaluating the utility of synthetic data, it does not fully reflect the movement characteristics of the intended elderly population. Testing on actual elderly falls is ethically and practically infeasible. Future work should focus on expanding the participant base and exploring methods to bridge this population gap through personalization and data-driven adaptation techniques.

## 6. Conclusions

Data scarcity poses a critical barrier to the development of robust fall detection systems, primarily due to the ethical and logistical constraints of collecting real-world fall events. To address this challenge, we propose two advanced synthetic data generation strategies: a Denoising Diffusion Probabilistic Model (DDPM) and a video-based pose estimation approach. These methods are systematically compared with conventional augmentation techniques, such as jittering, magnitude warping, and rotation. To elaborate, our Diffusion-based approach adopts the DDPM framework to generate synthetic fall data that closely mimics the statistical and temporal characteristics of real falls. On the other hand, the video extraction technique leverages the pose estimation technique from computer vision to capture fall events from videos and extract key motion patterns and sensor data. The distinct contribution of this work lies in the integration of the proposed methods, which are underexplored in fall detection, and their validation through both offline benchmarking and real-world (online) deployment. This dual validation ensures that the generated data not only aligns statistically with real-world signals but also translates effectively into practical, deployable fall detection systems.

In offline evaluations, across all datasets, including SmartFallMM, UniMiB, and K-Fall, the results showed that models trained with a mix of real and synthetic data outperformed those trained with real data alone, highlighting the value of synthetic data in enhancing model robustness and generalization. Specifically, the inclusion of DDPM-based and video-extracted synthetic data led to more accurate and reliable fall detection models.

Notably, our top-performing synthetic data generation methods for the SmartFallMM dataset, which are the Diffusion-based and video-extracted techniques, also demonstrated promising real-world performance, making it effective for practical deployment in fall detection systems. Specifically, the Diffusion-based method improved the offline F1-score on SmartFallMM by 11.11% (from 0.72 to 0.80), which corresponded to a 24% gain in the real-world F1-score (from 0.62 to 0.86) when tested using the SmartFall App. This strong alignment underscores the practical merit of offline evaluation as a reliable predictor of real-world performance.

## 7. Future Work

While our proposed methods demonstrated substantial improvements in both offline performance and real-world deployment, further enhancements are necessary for adoption in high-stakes environments, such as eldercare or clinical monitoring. To this end, our future work will focus on addressing the limitations by first exploring the personalization techniques that adapt the model to individual user behavior, the integration of additional sensor modalities (e.g., gyroscope, barometer, or ambient context), and the refinement of the synthetic data pipeline itself. In particular, based on our findings that the DDPM Diffusion-based and video extraction methods yielded the best results, we aim to refine the video extraction process by focusing on relevant frames only, as suggested by Votel and Li [37], who successfully used three frames per video for the training of a fall detection model. Extracting frames manually is time-consuming and labor-intensive, so we intend to explore automatic frame selection methods, such as the one proposed by Liu et al. [38], which automatically identifies images with fall activity. This approach could streamline the data preprocessing phase and potentially enhance the performance of the fall detection system by using only the most pertinent information from each video.

Secondly, we aim to further explore other video extraction techniques for generating synthetic fall data. Recording the real-world fall data from older adults poses significant challenges because of privacy issues, making it essential to explore alternative data sources. One such source is generative AI tools like Sora [39], an AI platform with text-to-video capabilities, which can produce videos up to one minute long. By leveraging videos of elderly individuals falling from AI tools, we can further enhance our synthetic datasets and improve fall detection models.

In parallel, we intend to explore the cross-modal data generation techniques such as text-to-motion methods IMUGPT [40] and T2M-GPT [41], which generate skeleton data-based motions from given text prompts. Those cross-model generation techniques are built upon a large human motion dataset.

Furthermore, future research could explore integrating fall-type classification into the synthetic data pipeline and extending these techniques, originally developed for fall detection, to other domains, such as sports injury prevention and rehabilitation monitoring. This could broaden the research’s impact by enhancing predictive models and monitoring systems across diverse movement-related applications.

Finally, while our study focuses on synthetic data generation using Diffusion models and video-based pose estimation, we acknowledge the critical role of gold-standard datasets based on 3D motion capture and artificially induced falls using programmable systems, like Motek. Such systems, equipped with built-in safety mechanisms, have recently enabled the collection of high-fidelity fall data through controlled perturbations. We believe that incorporating such gold-standard benchmarks will be essential in future studies to validate and further refine synthetic data pipelines, offering a complementary path alongside generative AI approaches.

## Figures and Tables

**Table 1:** Comparison of traditional, video-based, and AI-based synthetic data generation methods.

Method Type	Techniques Used	Key Features	Advantages	Limitations
Traditional Data Augmentation	(1) Jittering (2) Magnitude Warping (3) Rotation	(1) Applies noise, scaling, and rotation (2) Simulates signal variation (3) Enhances model robustness	(1) Simple and computationally efficient (2) Fast to integrate into training (3) Improves generalization to small perturbations	(1) Limited diversity in output (2) Cannot synthesize new or unseen fall dynamics
Video-Based Pose Extraction	(1) 2D-to-3D keypoint estimation (2) Derivation of velocity and acceleration signals	(1) Converts video motion into sensor-like signals (2) Captures fall dynamics from real-world footage	(1) Bridges vision and sensor modalities (2) Generates realistic joint-level signals (3) Cost-effective alternative to sensor data collection	(1) Manual labeling effort required (2) Privacy concerns in video sourcing (3) Background clutter may affect accuracy
DDPM-Based Synthetic Generation	(1) Diffusion models using U-Net (2) Score-based generative training	(1) Learns temporal patterns in real data (2) Generates high-fidelity synthetic sequences	(1) Produces diverse and naturalistic data (2) Captures subtle temporal variations (3) Supports privacy-preserving data synthesis	(1) High computational resource demands (2) Requires careful hyperparameter tuning

**Table 2:** Comparison of evaluation metrics for different methods across multiple datasets. Bold values indicate the best (lowest) score achieved for each metric and dataset combination.

Evaluation Metrics	Methods	SmartFallMM	UniMiB	K-Fall
Context-FID Score (↓)	Proposed Diffusion	**0.409**	**1.523**	**2.753**
	Diffusion-TS	0.722	2.192	3.179
	Video Extracted	0.910	2.654	3.854
	Jit	0.845	2.500	3.620
	MW	0.872	2.712	3.730
	Ro	0.801	2.410	3.500
Discriminative Score (↓)	Proposed Diffusion	0.704	1.620	**0.500**
	Diffusion-TS	**0.500**	**0.321**	1.155
	Video Extracted	0.910	1.875	0.882
	Jit	0.833	1.743	0.921
	MW	0.801	1.692	0.879
	Ro	0.850	1.730	0.901
Predictive Score (↓)	Proposed Diffusion	**0.735**	**0.745**	**0.759**
	Diffusion-TS	0.773	0.976	2.636
	Video Extracted	0.862	1.112	1.983
	Jit	0.820	1.045	1.901
	MW	0.793	1.089	2.001
	Ro	0.834	1.099	2.090
JSD (↓)	Proposed Diffusion	**0.056**	**0.009**	1.610
	Diffusion-TS	0.241	0.874	**0.458**
	Video Extracted	0.342	1.020	0.602
	Jit	0.295	0.981	0.598
	MW	0.310	1.001	0.610
	Ro	0.280	0.998	0.590
KS Score (↓)	Proposed Diffusion	**0.142**	**0.095**	0.345
	Diffusion-TS	0.231	0.112	0.347
	Video Extracted	0.150	0.103	**0.321**
	Jit	0.314	0.112	0.434
	MW	0.211	0.105	0.377
	Ro	0.188	0.121	0.459

**Table 3:** Fall detection model performance with synthetic data from various generation techniques across three datasets. Bold and italic values represent the highest and second-highest improvements observed in the fall detection task.

Datasets	Precision	Recall	F1-Score	Accuracy
**SF**	0.79	0.68	0.72	0.77
**SF w DF**	0.82	0.81	*0.80*	*0.82*
**SF w VE**	0.95	0.78	**0.86**	**0.88**
**SF w Jit**	0.78	0.66	0.73	0.71
**SF w MW**	0.82	0.64	0.72	0.71
**SF w Ro**	0.75	0.68	0.70	0.70
**UM**	0.71	0.75	0.74	0.76
**UM w DF**	0.79	0.83	*0.84*	*0.82*
**UM w VE**	0.82	0.88	**0.86**	**0.84**
**UM w Jit**	0.70	0.72	0.74	0.76
**UM w MW**	0.73	0.76	0.75	0.76
**UM w Ro**	0.69	0.72	0.71	0.73
**KF**	0.90	0.60	0.74	0.80
**KF w DF**	0.92	0.82	**0.86**	**0.88**
**KF w VE**	0.90	0.78	0.83	0.85
**KF w Jit**	0.80	0.78	0.79	0.80
**KF w MW**	0.85	0.72	0.80	0.81
**KF w Ro**	0.82	0.81	0.81	0.82

**Table 4:** Real-world results using SmartFFall App. Bold values represent the highest gain in the performance.

Dataset	Precision	Recall	F1-Score
SF	0.56	0.75	0.62
SF w DF	0.78	0.97	**0.86**
SF w VE	0.72	0.95	**0.82**

## Data Availability

We are happy to share the synthetic fall data generated in this study for research purposes upon request.
